# The influence of mutant genes APP and PSEN1 on hippocampal asymmetry in 5xFAD mice, transgenic model of Alzheimer disease

**DOI:** 10.18699/vjgb-26-92

**Published:** 2026-10

**Authors:** E.Yu. Brusentsev, I.N. Rozhkova, V.S. Kozeneva, I.D. Chekashov, T.A. Rakhmanova, O.B. Shevelev, S.Ya. Amstislavsky

**Affiliations:** Institute of Cytology and Genetics of the Siberian Branch of the Russian Academy of Sciences, Novosibirsk, Russia; Institute of Cytology and Genetics of the Siberian Branch of the Russian Academy of Sciences, Novosibirsk, Russia; Institute of Cytology and Genetics of the Siberian Branch of the Russian Academy of Sciences, Novosibirsk, Russia Novosibirsk State University, Novosibirsk, Russia; Institute of Cytology and Genetics of the Siberian Branch of the Russian Academy of Sciences, Novosibirsk, Russia Novosibirsk State University, Novosibirsk, Russia; Institute of Cytology and Genetics of the Siberian Branch of the Russian Academy of Sciences, Novosibirsk, Russia Novosibirsk State University, Novosibirsk, Russia; Institute of Cytology and Genetics of the Siberian Branch of the Russian Academy of Sciences, Novosibirsk, Russia; Institute of Cytology and Genetics of the Siberian Branch of the Russian Academy of Sciences, Novosibirsk, Russia

**Keywords:** Alzheimer’s disease, 5xFAD transgenic mice, APP and PSEN1 genes, hippocampus, dentate gyrus, diffusion tensor imaging, immunohistochemistry, neuroinflammation, beta-amyloid, neurogenesis, болезнь Альцгеймера, трансгенные мыши 5xFAD, гены APP и PSEN1, гиппокамп, зубчатая извилина, диффузионно-тензорная визуализация, иммуногистохимия, нейровоспаление, бета-амилоид, нейрогенез

## Abstract

Alzheimer’s disease (AD) is an age-related neurodegenerative pathology and one of the primary causes of dementia associated with memory and cognitive impairment. The dentate gyrus (DG) of the hippocampus plays a crucial role in learning and memory encoding, and it undergoes significant changes in AD. To study the underlying mechanisms of AD and to test new therapeutic agents, mouse models of this pathology are developed. One of the most popular and relevant models for AD research is the 5xFAD transgenic mouse strain carrying mutant human genes, specifically: amyloid beta precursor protein (APP) and presenilin-1 (PSEN1). The aim of this study was to investigate the influence of mutant APP and PSEN1 transgenes on the microstructure of the hippocampal DG in six-month-old heterozygous male 5xFAD transgenic mice using diffusion tensor magnetic resonance imaging (DTI-MRI) combined with immunohistochemical analysis. A special emphasis was placed on assessing hippocampal asymmetry, which had not been previously investigated in 5xFAD mice. In the hippocampal DG of both hemispheres in male 5xFAD mice, an elevated level of beta-amyloid was detected compared to the wild-type control group, combined with a higher density of microglia, but no differences were found in the level of neurogenesis. Along with this, hippocampal asymmetry was discovered in male 5xFAD mice, manifesting as an increased volume of the right hippocampus, a decreased level of fractional anisotropy in the right hippocampal DG, and a reduced neuron density in the left DG compared to the wild-type. Based on our results, it can be concluded that mutant human APP and PSEN1 genes cause alterations in the hippocampal DG, which may open new possibilities for the early diagnosis of Alzheimer’s disease. The identified changes in hippocampal volume and its asymmetry could become important biomarkers characterizing this pathology.

## Abstract (ru)

Влияние мутантных генов APP и PSEN1 на асимметрию гиппокампа у мышей 5xFAD – трансгенной модели болезни Альцгеймера

## Introduction

Alzheimer’s disease (AD) is an age-related pathology and one of the primary causes of dementia associated with memory and cognitive impairment (Bermejo-Pareja, Del Ser, 2024; Nasb et al., 2024; Perneczky et al., 2024). Human AD is characterized by the abnormal accumulation of extracellular beta-amyloid and intracellular hyperphosphorylated tau protein (Koss et al., 2016; Wang L. et al., 2016; Nasb et al., 2024). Genetic studies have demonstrated that these brain changes, which precede dementia and other symptoms of AD, can be linked to mutations in the gene encoding the amyloid precursor protein (Amyloid Precursor Protein – APP) and the gene encoding presenilin-1 (presenilin-1 – PSEN1) (De Strooper et al., 2012; Pink et al., 2012; Petit et al., 2022). The formation of beta-amyloid plaques, which begins years before the onset of major clinical symptoms, is considered one of the earliest signs of AD pathology (Bloom, 2014; Bermejo-Pareja, Del Ser, 2024). The accumulation of beta-amyloid, as well as the resulting neuroinflammation in the central nervous system (CNS), serve as important biomarkers both for monitoring the progression of AD (Adlard et al., 2014; Long, Holtzman, 2019; Perneczky et al., 2024) and for tracking the outcomes of therapeutic interventions aimed at mitigating this disease (Perneczky et al., 2024).

Volumetric magnetic resonance imaging (MRI) methods detect functional and structural brain abnormalities, including those in AD patients (Roe et al., 2021; Wang C. et al., 2022; Ai et al., 2025). A characteristic feature of AD is the disruption of white matter fiber integrity in the brain, which may be identified using diffusion tensor magnetic resonance imaging (DTI-MRI) (Rose et al., 2000). The DTI-MRI method is more sensitive compared to volumetric magnetic resonance imaging and is widely used to detect microstructural brain changes, particularly for assessing white matter integrity (Rose et al., 2000; Wang N. et al., 2019; Harrison et al., 2020; Veale et al., 2021).

Previously, using DTI-MRI, a decrease in fractional anisotropy (FA) was detected in certain brain regions of individuals suffering from AD, specifically in the corpus callosum, medial and temporal lobes, fornix, cingulate gyrus, precuneus, and the white matter of the prefrontal cortex (Teipel et al., 2012). Moreover, DTI-MRI has been used to identify microstructural changes in specific subregions of the hippocampus (Beaujoin et al., 2018; Shih et al., 2023). Analysis of these studies confirms that this method is promising for the early detection and monitoring of changes in distinct brain structures during Alzheimer’s disease.

To study the mechanisms underlying the development of the pathology and to test pharmaceuticals intended for the treatment of AD, animal models of this disease are applied (Jankowsky, Zheng, 2017; Sasaguri et al., 2017). In particular, one of the most popular models is the 5xFAD transgenic mouse strain (Research Models 5xFAD (C57BL6)), generated at The Jackson Laboratory (USA) on a C57BL/6 background. 5xFAD mice express human APP (with mutations: K670N/M671L, I716V, and V717I) and PSEN1 (with mutations: M146L and L286V) (Oakley et al., 2006; Oblak et al., 2021). Previously, using manganese-enhanced MRI, it was demonstrated that mutant human APP and PSEN1 genes alter regional brain activity in 5xFAD mice (Tang et al., 2016; Nie et al., 2019), while the application of DTI-MRI revealed a longer characteristic path length in weighted structural and functional elements of the connectome at minimum density (Kesler et al., 2018).

Mutant human APP and PSEN1 genes induce various brain abnormalities in transgenic 5xFAD mice depending on the homozygosity/heterozygosity of the strain (Richard et al., 2015). Amyloid precursor protein in pyramidal neurons of homozygous 5xFAD mice is formed as early as 16 days of age, and intraneuronal beta-amyloid accumulates by the age of 1.5 months; however, in heterozygous 5xFAD mice, beta-amyloid is detected at a later age (Richard et al., 2015).

Along with beta-amyloid accumulation, mutant human APP and PSEN1 genes cause abnormalities in the myelin sheaths of brain neurons in transgenic 5xFAD mice, and these pathological changes exacerbate as the animals ageing (Gu et al., 2018). Impairment of nerve fiber myelination, detectable particularly via MRI methods, is a critical marker of AD that manifests in the initial stages of the disease and represents a significant pathophysiological factor of this pathology (Butt et al., 2019; Bermejo-Pareja, Del Ser, 2024).

The hippocampus – a brain region playing a vital role in various processes, including learning, as well as memory formation and consolidation – undergoes substantial structural, morphological, and electrophysiological changes during aging and in neurodegenerative diseases such as Alzheimer’s disease (Padurariu et al., 2012; Bartsch, Wulff, 2015). In patients with early symptoms of AD, atrophy of the hippocampal dentate gyrus was identified, indicating the role of this brain structure in the development of this pathology (Hari et al., 2024).

To date, studies investigating the hippocampal dentate gyrus of 5xFAD mice have not accounted for hemispheric asymmetry (Giannoni et al., 2016; Kantarci et al., 2020; Nairuz et al., 2024; Zhu, Liu, 2025). The aim of this study was to investigate the influence of mutant human APP and PSEN1 genes on the microstructure of the dentate gyrus (DG region) of the hippocampus, as well as to evaluate hippocampal asymmetry in heterozygous mice of the 5xFAD transgenic strain using DTI-MRI and immunohistochemistry (IHC).

## Materials and methods

**Animals.** Five male 5xFAD mice (heterozygous mice modeling AD) and five wild-type (WT) males were investigated in the experiment. The animals were housed in the SPF vivarium of the Institute of Cytology and Genetics, SB RAS (Novosibirsk, Russia) in individually ventilated OptiMice cages (AnimalCare, USA) measuring 34.3 × 29.2 × 15.5 cm, at a temperature of 22–24 °C and a humidity of 40–50 %, with an inverted 12:12-hour light-dark cycle (lights on at 3:00 AM); fractional birch chips for laboratory animals (TU 16.10.23- 001-0084157135-2019) were used as bedding. The animals had ad libitum access to autoclaved standardized chow for laboratory rats and mice “Delta Feeds” LbK 120 R-22, GOST 34566-2019 (BioPro, Russia) and purified water enriched with mineral supplements.

**Experimental design.** The graphical layout of the experimental design is presented in Figure 1.

**Fig. 1.**
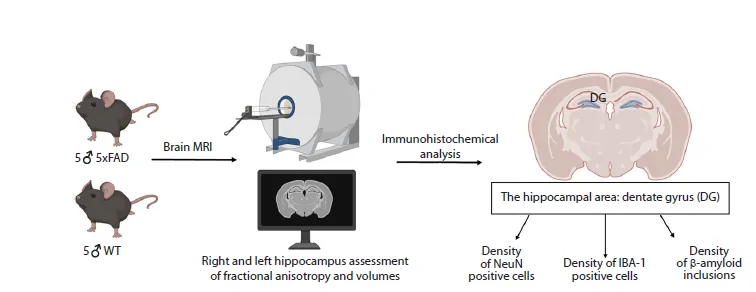
The graphical layout of the experimental design. WT – wild type.

**Diffusion tensor magnetic resonance imaging.** Assessment of brain anatomy, as well as the continuity and density of nerve fibers in the hippocampus of 5xFAD and WT mouse strains, was performed using MRI according to protocols described previously (Naumenko et al., 2018; Lipengolts et al., 2022; Zhang C. et al., 2023). Mice were anesthetized using a mixture of oxygen and isoflurane (98.5/1.5 %) and then placed into the MR scanner on a specialized platform. Prior to scanning, each animal was secured on a custom cradle, after which a respiration sensor was attached to monitor the depth of anesthesia.

Magnetic resonance imaging was performed for 20–30 minutes per animal on a ClinScan 7T experimental scanner for small animals (Bruker BioSpin, Germany) using a volume mouse head coil and the following pulse sequences: 1) fast T1-weighted images based on a gradient echo pulse sequence in three orthogonal planes, repeated at least three times or until correct anatomical sections are obtained through the brain structure for sequential transmission of slices of the main pulse sequences; 2) a diffusion-weighted pulse sequence with b-factors of 0 and 1,200 s/mm2, 64 diffusion directions, TE = 32 ms, TR = 8,000 ms, 4 averages, voxel size of 0.163542 × 0.163542 × 0.8 mm3 (FOV = 13 × 15 mm) without an interslice gap, and images with the same resolution at a b-factor of 0 s/mm2 with a 180-degree phase-encoding gradient reversal; 3) anatomical T2-weighted images with the following parameters: TE = 80 ms, TR = 3,430 ms, ETL = 14, voxel size of 0.083 × 0.083 × 0.8 mm3 (FOV = 16 × 16 mm) without an interslice gap.

The obtained data processing was performed in the DSIStudio software package using the following steps: 1) data conversion into the NIfTI (Neuroimaging Informatics Technology Initiative) format; 2) TOPUP and EDDY inhomogeneity correction; 3) motion artifact correction; 4) data reconstruction using two methods: Generalized q-sampling imaging – GQI (Yeh et al., 2010) and Q-space diffeomorphic reconstruction – QSDR (Yeh, Tseng, 2011). For GQI, interpolation to an isotropic voxel of 0.1635 and reconstruction with the preservation of the original spatial orientation were performed. For QSDR, spatial orientation alignment of the images and reconstruction to the mouse atlas with an isotropic voxel of 0.1630 were conducted; 5) export of fractional anisotropy (FA) maps; 6) loading the DukeRCCF atlas (Johnson et al., 2022) and maps into a Python script; 7) calculation of the median, first, and third quartiles in target zones of the selected atlas via its registration onto the obtained FA maps using both methods (GQI and QSDR).

**Hippocampal volume assessment.** The volumes of the right and left parts of the hippocampus were calculated as the sum of the areas of the structures on brain slices obtained from T2-weighted images, multiplied by the slice thickness, using the ImageJ software (Zapara et al., 2018).

**Intracardiac perfusion.** Perfusion was performed through the circulatory system for brain tissue fixation, as described previously (Rozhkova et al., 2023). Immediately after completion of the MRI, mice were anesthetized with an intramuscular injection of 75 μL (per 10 g of body weight) of medetomidine hydrochloride (Meditin, 1 mg/mL; Api-San, Russia) and 60 μL (per 10 g of body weight) of Zoletil (Virbac, France). Next, 30–50 mL of phosphate-buffered saline (PBS) followed by 30–50 mL of a 10 % formalin solution prepared in PBS were infused through the circulatory system. Thereafter, the brains were harvested and stored in a 30 % sucrose solution prepared in PBS containing 10 % formalin at 4 °C for dehydration and subsequent fixation over the next two weeks until the tissue sank to the bottom of the container. Brain samples were embedded in Tissue-Tek O.C.T. (Sakura Finetek, USA), then frozen and stored at –70 °C.

**Preparation of frozen brain slices. **In accordance with the atlas coordinates (Paxinos et al., 2012), frozen sections of the left and right hippocampus (including the dentate gyrus – DG) were cut at a distance of –1.46 (–1.82) mm from bregma. Sections with a thickness of 20 μm were prepared at –25 °C using an HM550 OP cryostat (Thermo Scientific, USA) and placed on PCI adhesive-coated microscope slides with ground edges and a colored frosted end (CITOTEST, China).

**Immunohistochemical staining.** Sample staining was performed according to the manufacturers’ kit protocols with minor modifications (Rozhkova et al., 2023). Prior to the staining procedure, sections were dehydrated followed by rehydration for five minutes in PBS. Then, after rehydration, heat-induced epitope retrieval was performed in 10 mM alkaline citrate buffer (pH = 9) at 95 °C in a TW-2.02 water bath (Elmi, Latvia) for 10 min. Following this, the sections were removed from the buffer and cooled to room temperature. The samples were then washed three times in PBS-Tween buffer: PBS supplemented with 0.1 % Tween-20 P9416-100ML (Merck, Germany). Next, ProteinBlock (ab64226; Abcam, UK) was added to each section for 30 min, and excess liquid was subsequently removed in accordance with the manufacturer’s recommendation. After that, 50 μL of the primary antibody was added, and the sections were incubated overnight at 4 °C in a humidified dark chamber.

The primary antibody concentrations used were 1:750 for anti-NeuN (ab177487, Abcam, UK), 1:500 for AIF1/ IBA1 Rabbit mAb (A19776, ABclonal, Germany), 1:500 for β-Amyloid(1-42) Rabbit mAb (A24422, ABclonal, Germany), and 1:750 for anti-DCX (ab18723, Abcam, UK). Sections were then washed with PBS-Tween buffer, excess liquid was removed, and 50 μL of secondary antibody Goat Anti-Rabbit IgGH & LAF488 (ab150077, Abcam, UK) at a 1:650 dilution was applied. Sections were incubated in a dark humidified chamber for two hours at 4 °C. Following incubation, the samples were washed with PBS-Tween buffer, excess fluid was removed, and sections were mounted on microscope slides in ProLong Glass Antifade Mountant (Thermo P36982, Thermo Fisher Scientific, USA).

To identify cell nuclei, sections stained with AIF1/IBA1 Rabbit mAb and β-Amyloid (1-42) Rabbit mAb were additionally treated with 80 μL of DAPI (Thermo Fisher Scientific, USA) at a concentration of 2 mg/mL, incubated in a dark humidified chamber for 15 minutes, and then washed twice in PBS for 3 min before applying the ProLong Glass Antifade Mountant.

**Neuronal density analysis.** Analysis of the density of antibody-labeled neurons in the DG region of the hippocampus was performed using a ZEISS AxioImager 2 upright fluorescent microscope (Carl Zeiss, Germany). The average number of antibody-labeled cells in the region of interest was calculated from at least four sections for each animal. Evaluation of the number of granular and DCX-positive cells was carried out in the granular layer of the DG. Beta-amyloid inclusions and IBA1-positive cells were counted simultaneously in two layers of the DG (granular and polymorphic). To evaluate the density of DCX-positive cells, beta-amyloid inclusions, and IBA1-positive cells, a field of view corresponding to the size of the studied area was used; to evaluate the density of NeuNpositive neurons, at least three fields of view (2,000 μm2) on a single image were used (Zhang L. et al., 2021). ImageJ software was used to create the field of view. The density (millions of cells, n × 105) in volume (mm3) was calculated based on the average number of cells in the region, as described previously (Rozhkova et al., 2023).

**Statistical analysis. **Data analysis was performed using the STATISTICA v. 12.0 software package (StatSoft, Inc., USA). All data were tested for normality using the Shapiro–Wilk W-test. To evaluate the impact of the “hemispheric asymmetry” factor on the level of fractional anisotropy, hippocampal volumes, as well as the density of neurons, microglia, DCX, and beta-amyloid inclusions in the DG region of the hippocampus, the Wilcoxon signed-rank test was used, followed by pairwise comparisons between groups of different genotypes using the Mann–Whitney U-test. To evaluate the density of neurons, microglia, DCX, and beta-amyloid inclusions, the Kruskal–Wallis H-test was additionally applied for comparison based on the “genotype” factor. Data are presented as median with the first and third quartiles – Me [Q1;Q3]. The level of statistical significance was set at p < 0.05.

## Results

Data on hippocampal volume are presented in Figure 2. Statistical analysis using the Wilcoxon test showed no effect of the “hemispheric asymmetry” factor [T = 23; p > 0.05] on this parameter. Nevertheless, the Mann–Whitney U-test revealed an effect of the “genotype” factor (U = 1; |Z| = 2.30; p < 0.05), specifically, a significantly larger hippocampal volume in 5xFAD mice than in WT mice (13.599 [13.583;14.834] vs. 12.445 [11.556;12.622] μL3, respectively) in the right hemisphere of the brain.

**Fig. 2.**
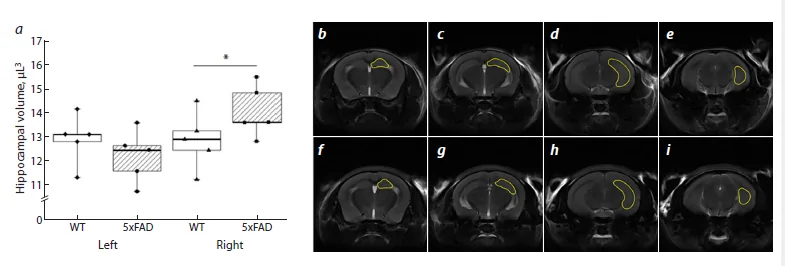
Hippocampal volume in 5xFAD and WT mouse strains. a – volumes of the left and right hippocampi of 5xFAD and WT mice; b–e – T2-weighted images of hippocampal slices of 5xFAD mice in the right hemisphere of the brain; f–i – T2-weighted images of hippocampal slices of WT mice in the right hemisphere of the brain. * p < 0.05 between WT and 5xFAD.

Data on FA in the hippocampal DG are presented in Figure 3. The Wilcoxon test showed a significant effect of the “hemispheric asymmetry” factor [T = 2; p < 0.01] on FA in the DG region of the hippocampus. Comparison using the Mann– Whitney U-test revealed an effect of the “genotype” factor on the FA level (U = 2; |Z| = 2.09; p <0.05) in the hippocampal DG region; FA was lower on the right side in 5xFAD mice compared to control WT mice (0.106 [0.010;0.115] vs. 0.126 [0.124;0.143], respectively) (Fig. 3a).

**Fig. 3.**
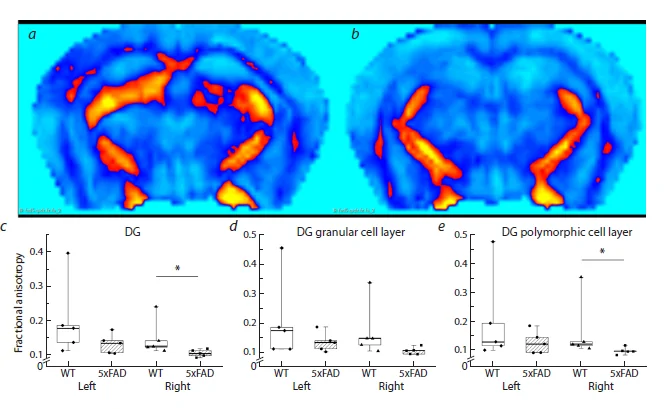
Fractional anisotropy (FA) levels in the hippocampal DG region of WT (a) and 5xFAD (b) mouse strains. c – FA in the DG; d – FA in the granular layer of the DG; e – FA in the polymorphic layer of the DG. * p < 0.05 between WT and 5xFAD.

Statistical analysis using the Wilcoxon test demonstrated an effect of the “hemispheric asymmetry” factor [T = 5; p < 0.05] on FA in the granular layer of the hippocampal DG region. However, comparison using the Mann-Whitney U-test revealed no effect of the “genotype” factor either for the left hemisphere ([U = 10; |Z| = 0.42; p > 0.05]) or for the right hemisphere ([U = 3; |Z| = 1.89; p > 0.05]) in the granular layer of the hippocampal DG (Fig. 3b). The Wilcoxon test showed no significant effect of the “hemispheric asymmetry” factor [T = 10; p > 0.05] on FA in the polymorphic layer of the hippocampal DG region. Comparison using the Mann– Whitney U-test revealed an effect of the “genotype” factor on FA (U = 2; |Z| = 2.09; p < 0.05) in the polymorphic layer of the hippocampal DG region on the right side in 5xFAD mice compared to WT (0.096 [0.096;0.098] vs. 0.121 [0.116;0.130], respectively) (Fig. 3c).

Data on immunohistochemical staining are presented in Figures 4–6. Differences in total neuronal density within the granular layer of the DG are presented in Figure 4. Statistical analysis using the Wilcoxon test revealed no effect of the “hemispheric asymmetry” factor [T = 10; p > 0.05] on neuronal density in the granular layer of the DG. Analysis using the Kruskal–Wallis H-test demonstrated an effect of the “genotype” factor [H(1.20) = 7.20; p < 0.01] on this parameter. Furthermore, comparison using the Mann–Whitney U-test revealed that within the granular layer of the DG in 5xFAD males, neuronal density in the left hemisphere was significantly lower (U = 2.5; |Z| = 1.98; p <0.05) than in WT (10.15 [9.69;10.17] vs. 10.95 [10.70;11.21], respectively) (Fig. 4f ).

**Fig. 4.**
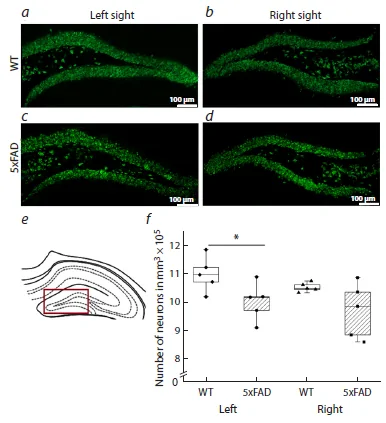
Neuronal density in the granular layer of the DG; neurons are labeled with antibodies against the neuronal marker (NeuN). a–d – microphotographs of DG sections; a – left hemisphere of the brain of wild-type (WT) males; b – right hemisphere of the brain of wild-type (WT) males; c – left hemisphere of the brain of 5xFAD males; d – right hemisphere of the brain of 5xFAD males; e – schematic representation of the studied hippocampal region; f – number of neurons per mm3. Magnification 400х. * p < 0.05 between WT and 5xFAD in the left hemisphere of the brain.

**Fig. 5.**
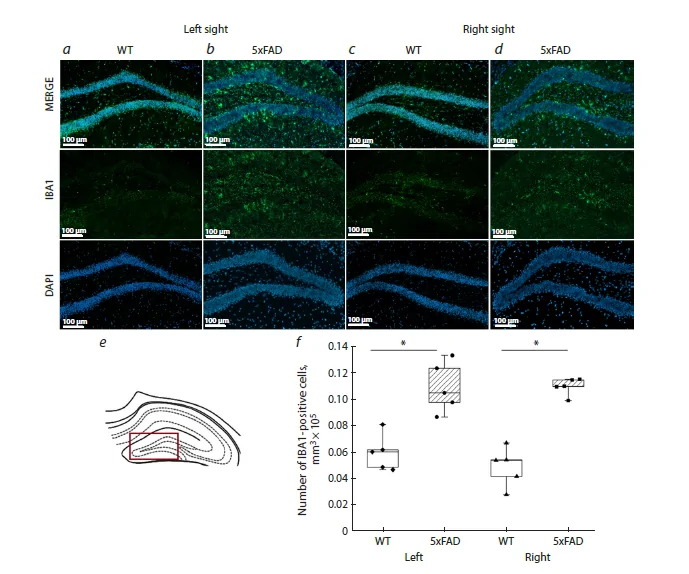
Microglia density in the DG; cells are labeled with antibodies against IBA1 (ionized calcium-binding adapter molecule 1); cell nuclei are counterstained with DAPI. a–d – microphotographs of DG sections; a – left hemisphere of the brain of wild-type (WT) males; b – left hemisphere of the brain of 5xFAD males; c – right hemisphere of the brain of wild-type (WT) males; d – right hemisphere of the brain of 5xFAD males; e – schematic representation of the studied hippocampal region; f – number of IBA-1 positive cells per mm3. Magnification 200х. * p < 0.05 between WT and 5xFAD in the left hemisphere of the brain.

**Fig. 6.**
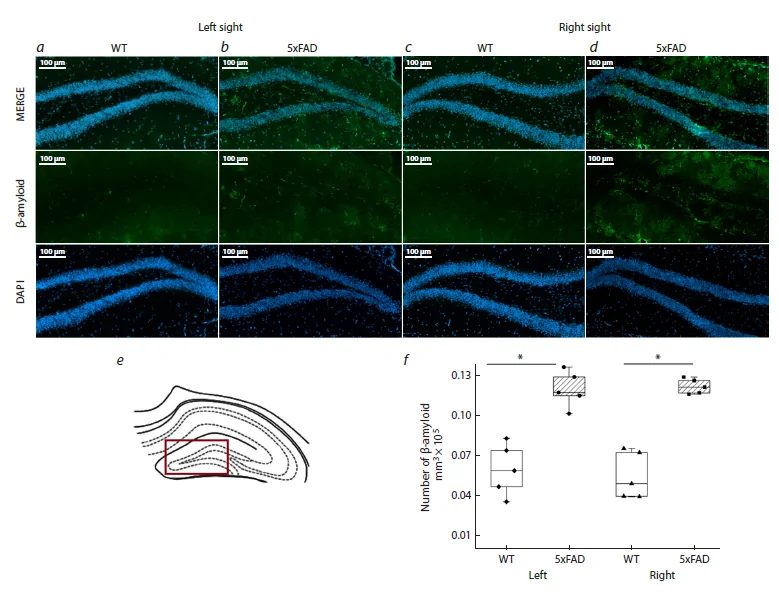
Density of beta-amyloid inclusions in the DG; beta-amyloid is labeled with antibodies against β-amyloid (1-42); cell nuclei are counterstained with DAPI. a–d – microphotographs of DG sections; a – left hemisphere of the brain of wild-type (WT) males; b – left hemisphere of the brain of 5xFAD males; c – right hemisphere of the brain of wild-type (WT) males; d – right hemisphere of the brain of 5xFAD males; e – schematic representation of the studied hippocampal region; f – number of beta-amyloid inclusions per mm3. Magnification 200х. * p <0.05 between WT and 5xFAD.

Statistical analysis using the Wilcoxon test revealed an effect of the “hemispheric asymmetry” factor [T = 2; p < 0.01] on the level of neurogenesis in the granular layer of the DG. However, the Kruskal–Wallis H-test showed no effect of the “genotype” factor [H(1.20) = 1.46; p > 0.05] on this parameter. Comparison using the Mann–Whitney U-test also revealed no differences ( p > 0.05) in this parameter between 5xFAD and WT males in the hippocampal dentate gyrus either on the left (0.05 [0.04;0.05] vs. 0.05 [0.05;0.06], respectively) or on the right (0.05 [0.04;0.05] vs. 0.05 [0.04;0.05], respectively).

The results regarding the density of IBA1-expressing cells in the granular and polymorphic layers of the DG are presented in Figure 5. Statistical analysis using the Wilcoxon test revealed no significant effect of the “hemispheric asymmetry” factor [T = 22; p > 0.05] on the density of IBA1-expressing cells. Nevertheless, the Kruskal–Wallis H-test demonstrated an effect of the “genotype” factor [H(1.20) = 13.50; p <0.001] on this parameter. Comparison using the Mann–Whitney U-test revealed that within the DG, 5xFAD mice exhibited a higher density of IBA1-positive cells in both the left (U = 0; |Z| = 2.51; p< 0.05) and right hemispheres (U = 0; |Z| = 2.51; p <0.05) compared to control WT mice (0.10 [0.10;0.12] vs. 0.06 [0.05;0.06] for the left side; 0.11 [0.11;0.11] vs. 0.05 [0.04;0.05] for the right side, respectively) (Fig. 5f ).

Differences in the total density of beta-amyloid inclusions in the granular and polymorphic layers of the DG are presented in Figure 6. Statistical analysis using the Wilcoxon test revealed no effect of the “hemispheric asymmetry” factor [T = 19; p > 0.05] on the density of beta-amyloid inclusions in the DG. The Kruskal–Wallis H-test demonstrated an effect of the “genotype” factor [H(1.20) = 14.28; p < 0.001] on this parameter. Comparison using the Mann–Whitney U-test revealed that in the hippocampal dentate gyrus of 5xFAD mice, the density of beta-amyloid was higher than in the corresponding region of wild-type mice, both on the left (U = 0; |Z| = 2.51; p < 0.05) and on the right (U = 0; |Z| = 2.51; p < 0.05) compared to the corresponding region of wild-type mice (0.12 [0.12;0.13] vs. 0.06 [0.05;0.07] for the left side; 0.12 [0.12;0.13] vs. 0.05 [0.04;0.07] for the right side, respectively) (Fig. 6f ).

## Discussion

Mutant APP and PSEN1 genes induce beta-amyloid accumulation in the CNS, which is one of the most significant hallmark features of AD (Petit et al., 2022; Nasb et al., 2024; Perneczky et al., 2024). In our study, a substantial increase in beta-amyloid inclusions was detected in the hippocampal dentate gyrus of heterozygous 5xFAD mice carrying mutant human APP and PSEN1 genes in both the right and left hemispheres of the brain, which aligns with the results of previous studies demonstrating elevated beta-amyloid levels in the hippocampal DG of this AD model (Giannoni et al., 2016; Kantarci et al., 2020; Nairuz et al., 2024; Zhu, Liu, 2025).

In particular, a study by T. Nairuz et al. (2024) showed that at 6 and 12 months of age, the density of beta-amyloid inclusions in male 5xFAD mice was significantly increased in the CA1, CA3, and DG regions of the hippocampus. Furthermore, at six months of age, among the three investigated hippocampal subfields, the maximum level of beta-amyloid inclusions was observed precisely in the DG of 5xFAD mice compared to control wild-type mice (Nairuz et al., 2024). A recently published study demonstrated that in female 5xFAD mice at an even earlier age of four months, a similar increase in beta-amyloid inclusions was observed in the hippocampal dentate gyrus compared to control wild-type animals (Zhu, Liu, 2025). The accumulation of beta-amyloid in the frontal, parietal, entorhinal cortex, and the hippocampal dentate gyrus of 5xFAD mice is accompanied by blood vessel damage in these brain regions (Giannoni et al., 2016).

Beta-amyloid accumulation can induce neuroinflammation in various brain regions of AD patients (Long, Holtzman, 2019; Perneczky et al., 2024). Microglial cells are activated in response to beta-amyloid accumulation in the brain and cluster around beta-amyloid plaques (Meyer-Luehman et al., 2008).

In our study, a substantial increase in microglia density was detected in both the right and left parts of the hippocampal dentate gyrus in male 5xFAD mice carrying mutant human APP and PSEN1 genes, which aligns with the results of other studies that identified neuroinflammation in the hippocampus of 5xFAD mice starting from two months of age (Oakley et al., 2006; Kantarci et al., 2020; Nairuz et al., 2024; Westi et al., 2024). In particular, at six months of age, the level of neuroinflammation in the hippocampal subiculum of heterozygous 5xFAD mice significantly increases compared to wild-type littermates (Westi et al., 2024). A substantial increase in microglial density at 6–8 months of age is also noted in the hippocampal DG region of 5xFAD mice (Kantarci et al., 2020; Nairuz et al., 2024), which is in good agreement with our findings.

Previously, it was established that neurogenesis impairment occurs in the hippocampal DG region of AD patients (Moreno- Jimenez et al., 2019; Disouky, Lazarov, 2021). However, we did not detect any changes in the level of neurogenesis in the dentate gyrus of 5xFAD mice at six months of age. In studies conducted at 2–4 months of age, a decrease in the level of hippocampal neurogenesis was shown in 5xFAD mice compared to control wild-type animals, as well as its increase during experimental forms of treatment (Moon et al., 2014; Ding et al., 2023; Lee et al., 2025). However, by seven months of age in 5xFAD mice, differences in this parameter compared to wild-type littermates disappeared (Moon et al., 2014), which is possibly associated with a sharp decline in the level of neurogenesis in both 5xFAD mice and control wild-type mice

Neuronal loss is characteristic of the late stages of AD (Braak H., Braak E., 1991). Meanwhile, neuronal loss in AD is not uniform throughout the brain: the entorhinal cortex and certain subfields of the hippocampus prove to be the most vulnerable (Braak H., Braak E., 1991; Shi et al., 2009; Shih et al., 2023).

Similar changes have also been detected in 5xFAD mice, which exhibited an age-dependent decline in the total number of neurons across various hippocampal zones, specifically CA1, CA3, and the DG (Jawhar et al., 2012; Kim et al., 2019; Zhang L. et al., 2021; Francis et al., 2024). In the present study, it was established that in six-month-old 5xFAD males, there were fewer neurons in the hippocampal DG of the left hemisphere compared to control wild-type animals, whereas no such changes were detected in the DG of the right hemisphere.

The results of previous studies evaluating neuronal density in the hippocampus of 5xFAD mice are ambiguous. At 4.5 months of age, no significant difference from the control is found in the number of neurons within the hippocampal DG of 5xFAD mice (Kim et al., 2019), but a decrease in the number of neurons in this structure is observed at 6–14 months of age (Kim et al., 2019; Zhang L. et al., 2021; Francis et al., 2024).

In modern neurobiology, issues of brain lateralization and asymmetry are actively studied, as they can be crucial for understanding functional differences between the hemispheres and responses to pathology (Francis et al., 2024). The asymmetrical decrease in the number of neurons in the left DG of 5xFAD mice discovered by us is of particular interest, because previous studies aimed at investigating this hippocampal region in this AD model overlooked this factor and evaluated the number of neurons in the DG as a whole, without distinguishing between the right and left hemispheres of the brain (Kim et al., 2019; Zhang L. et al., 2021; Francis et al., 2024).

Atrophy of various hippocampal subfields and the hippocampus as a whole, detected by MRI methods, is an important diagnostic sign of Alzheimer’s disease (Shi et al., 2009; Jahanshahi et al., 2023; Hari et al., 2024). In patients with mild cognitive impairment, the volume of left hemisphere structures is typically altered less than that of the right hemisphere (right lateralization), although pronounced forms of AD exhibit a certain reduction in the prominence of right hippocampal lateralization (Shi et al., 2009; Jahanshahi et al., 2023).

In our study, it was revealed that mutant human APP and PSEN1 genes in 5xFAD mice can influence changes in hippocampal volume; the volume of the right hippocampus in 5xFAD mice was larger than that of control wild-type mice. Meanwhile, no differences in hippocampal volume were detected in the left hemisphere.

In studies conducted on 5xFAD mice at 2–6 months of age, no changes in hippocampal volume were found in comparison with control wild-type animals (Girard et al., 2014; Westi et al., 2024). However, at 13 months of age, the hippocampal volume in 5xFAD mice decreased (Macdonald et al., 2014). The authors of the aforementioned studies evaluated the hippocampal volume as a whole, without distinguishing right and left parts and without assessing its individual zones. Meanwhile, our results obtained from 5xFAD mice indicate that measuring hippocampal volume while accounting for asymmetry may serve as a more reliable marker of changes characterizing AD, which aligns with the conclusions of a meta-analysis of clinical data (Shi et al., 2009).

Previously, using DTI-MRI, a decrease in fractional anisotropy was detected in certain brain regions of individuals suffering from AD, specifically in the corpus callosum, medial and temporal lobes, fornix, cingulate gyrus, precuneus, and the white matter of the prefrontal cortex (Teipel et al., 2012). The decrease in FA may be associated with an expansion of the extracellular space volume and neurodegeneration (Hong et al., 2013; Nowrangi et al., 2013), and it can indicate that water molecules move not in a single direction, i. e., along axons as they normally should, but more uniformly across the entire area due to nerve fiber damage. The reduction in FA detected via MRI is considered an indicator of beta-amyloid accumulation (Maharjan et al., 2022).

Brain studies in mice using genetic models such as 3xTg and 5xFAD have revealed a decrease in FA in several brain structures (Snow et al., 2017; Maharjan et al., 2022). Changes in FA levels can occur differently depending on the studied brain region, which may be associated with its complex microstructure and heterogeneous accumulation of beta-amyloid, and could also be driven by the associated neuroinflammation (Praet et al., 2018; Maharjan et al., 2022).

In the present study, a decrease in FA was revealed in the hippocampal dentate gyrus of the right hemisphere in male 5xFAD mice compared to control wild-type animals. Our data indicating asymmetry in FA changes in the brain of 5xFAD mice are consistent with the observation that different mouse strains modeling AD have shown asymmetry in the development of this pathology between the hemispheres regarding changes in the levels of beta-amyloid accumulation and neuroinflammation levels (Biechele et al., 2021; Shipton et al., 2022; Morrissey et al., 2023).

Previously, no FA impairments were found in the hippocampus of 5xFAD mice (Maharjan et al., 2022). However, in that study, FA was evaluated within the hippocampus as a whole without distinguishing the left and right hemispheres and without subdividing it into regions. Our data indicate that evaluating FA in specific hippocampal regions, in the DG particularly, can be a more sensitive indicator of AD-characteristic brain impairments. Moreover, it is necessary to account for the asymmetry of the right and left hemispheres regarding this feature. Nevertheless, it must be acknowledged that the small sample size in the present study imposes limitations on the interpretation of the results and requires verification in larger cohorts.

The hippocampus is a critical brain structure where various types of impairments occur in AD (Moreno-Jimenez et al., 2019; Disouky, Lazarov, 2021; Zeng et al., 2021; Jahanshahi et al., 2023; Shih et al., 2023). The functional anatomy of hippocampal subfields, as well as the role of each subfield in the storage and processing of visual and verbal information, episodic memory, navigation, and object recognition, has been studied in detail (Bartsch, Wulff, 2015; Zammit et al., 2017; Pang et al., 2019; Zeng et al., 2021). Meanwhile, it has been shown that during the progression of AD, the rate and extent of impairments differ significantly across different hippocampal subfields (Padurariu et al., 2012; Bartsch, Wulff, 2015). It has been suggested that alterations in individual hippocampal regions may serve as a more precise marker for AD than changes evaluated in the hippocampus as a whole without subfield distinguishing (Zilioli et al., 2025).

The DG plays a special role in the neural processes underlying learning and memory; information encoding processes occur within this structure, and it receives input and output signals from various hippocampal regions (CA1–CA4) and the neocortex (Amaral et al., 2007; Jonas, Lisman, 2014; Bartsch, Wulff, 2015; Jahanshahi et al., 2023; Shih et al., 2023). Adult neurogenesis in humans occurs within the dentate gyrus, continuing into old age but becoming impaired as AD progressed (Moreno-Jimenez et al., 2019), which makes the hippocampal dentate gyrus of particular interest in AD research (Shih et al., 2023).

In the present study, six-month-old heterozygous male 5xFAD mice carrying mutant human APP and PSEN1 genes demonstrated a hemispheric asymmetry against a background of overall elevated beta-amyloid levels and neuroinflammation, alongside an unchanged level of neurogenesis in the hippocampal DG of both hemispheres. This asymmetry manifested as a difference in hippocampal volume between the right and left hemispheres, a decrease in FA within the right DG, and a reduction in neuronal density within the left DG.

Several studies have noted an asymmetry in the progression of features characteristic of AD between the brain hemispheres, particularly in the cortex (Derflinger et al., 2011; Roe et al., 2021), thalamus (McGeown et al., 2008), and hippocampus (Shi et al., 2009; Jahanshahi et al., 2023). Using volumetric MRI and subsequent analysis of T1-weighted images in AD patients, changes in the hippocampal asymmetry index (the ratio of right-to-left hippocampal volumes) were observed, with alterations being more pronounced on the right side than on the left in several of its subfields (Jahanshahi et al., 2023).

Previously, it was established that under normal conditions, mice and rats exhibit a pronounced structural and functional left-right asymmetry of the hippocampus (El-Gaby et al., 2015; Sakaguchi, Sakurai, 2020), which could also influence the differing levels of AD progression between hemispheres in male 5xFAD mice. M. El-Gaby et al. (2015) point out in their review that the hippocampi of the left and right hemispheres possess distinct functional lateralization, and they analyze the mechanisms underlying this asymmetry. The left hippocampus specializes in associative long-term memory, while the right specializes in spatial orientation. Such functional differentiation is achieved through structural differences at the synaptic level (El-Gaby et al., 2015).

In our study, the hippocampal asymmetry revealed for the first time in 5xFAD mice with mutant human APP and PSEN1 genes, in particular the difference in hippocampal volume between the right and left hemispheres of the brain, may be associated with the accumulation of beta-amyloid in the DG observed in 5xFAD mice and the resulting increase in neuroinflammation on both sides, combined with a reduction in neuronal density only in the DG of the left hippocampus. It is possible that in male 5xFAD mice, the volume of the left hippocampus does not differ from the wild type because the reduction in the number of neurons within its DG is counterbalanced by the overall increase in the density of beta-amyloid plaques and microglia, whereas the right hippocampus expanded due to the preservation of cell density and, potentially, an expansion of the extracellular space associated with neuroinflammation. A second hypothesis is that the hippocampal volume in both the right and left hemispheres could have been larger at an earlier age in male 5xFAD mice, than in control WT mice due to early beta-amyloid accumulation and subsequent neuroinflammation; however, the later decrease in the number of neurons within the left DG eliminated the difference with control animals specifically on the left side.

Some studies revealed an asymmetry in the development of pathological changes characteristic for AD between the brain hemispheres, particularly in the cortex (Derflinger et al., 2011; Roe et al., 2021), thalamus (McGeown et al., 2008), and hippocampus (Shi et al., 2009; Jahanshahi et al., 2023). Previously, it was hypothesized that during the progression of AD, the rate of atrophy across different regions of the right and left hippocampi may differ (Shi et al., 2009). The results of our study support this hypothesis.

## Conclusion

Based on our findings and the analysis of literature data, we believe that mutant human APP and PSEN1 genes induce alterations in the dentate gyrus (DG region) of the hippocampus, and evaluating the microstructure of this brain area may serve as an indicator of AD-characteristic impairments and in the future might be used for the early diagnosis of AD (Shih et al., 2023). In particular, the changes in hippocampal volume and its asymmetry identified in our study could become a useful biomarker for AD diagnosis.

**Compliance with ethical standards.** All studies were approved by the Bioethics Commission of the Institute of Cytology and Genetics SB RAS (Protocol No. 215 dated February 25, 2025) and comply with the European Convention for the Protection of Vertebrate Animals used for Experimental and Other Scientific Purposes (ETS No. 123).

## Conflict of interest

The authors declare no conflict of interest.
